# Enzootic calcinosis caused by *Solanum glaucophyllum* in cattle: retrospective analysis of 23 outbreaks in Central Argentina.

**DOI:** 10.29374/2527-2179.bjvm005425

**Published:** 2025-11-10

**Authors:** Emiliano Sosa, Germán José Cantón, Facundo Urtizbiria, Eleonora Morrell, María Valeria Scioli, Ernesto Odriozola, Juan Agustín García

**Affiliations:** 1 Instituto de Innovación para la Producción Agropecuaria y el Desarrollo Sostenible (IPADS), INTA Balcarce-CONICET, Balcarce 7620, Buenos Aires, Argentina

**Keywords:** Solanum glaucophyllum, beef cattle, systemic mineralization, ruminants, toxic plants, Solanum glaucophyllum, gado de corte, mineralização sistêmica, ruminantes, plantas tóxicas

## Abstract

Enzootic calcinosis (EC) is a chronic disease mainly affecting ruminants consuming calcinogenic plants. In Argentina, EC is associated by the consumption of *Solanum glaucophyllum* in beef grazing cattle and is one of the most frequent toxicities affecting livestock from low-flooded areas. In this paper, we describe 23 outbreaks of EC in beef cattle due to consumption of *S. glaucophyllum* in central Argentina between 1990 to 2024. Outbreaks occurred more frequently during the summer-autumn (February-May) and affected more frequently adult beef cattle. An average morbidity and mortality of 13.75% and 4.22% were registered, respectively. The main clinical signs were progressive emaciation, limbs stiffness, and lameness. Necropsies were performed, and gross findings included multifocal-coalescent mineralization of blood vessels, heart, and lung. Microscopically, severe diffuse mineralization was observed mainly in the endocardium and intima and media layers of the aorta. Intra-alveolar mineralization was observed lining the wall of the alveolar septa, confirming calcium salt deposits by von Kossa staining. Blood calcium and phosphorus values in affected cattle remained within the reference ranges. Consumption of *S. glaucophyllum* was confirmed in all outbreaks. Despite being an endemic problem in the beef industry in Argentina, there are no effective treatments or control strategies, therefore, further studies are necessary to prevent the occurrence of EC.

## Resumo

Calcinose enzoótica (CE) é uma doença crônica que afeta principalmente ruminantes que consomem plantas calcinogênicas. Na Argentina, a CE está associada ao consumo de *Solanum glaucophyllum* em gado de corte e é uma das toxicidades mais frequentes que afetam o gado de áreas de baixa inundação. Neste artigo, descrevemos 23 surtos de CE em bovinos de corte devido ao consumo de *S. glaucophyllum* na Argentina central entre 1990 e 2024. Os surtos ocorreram com mais frequência durante o verão-outono (fevereiro-maio) e afetaram mais frequentemente o gado bovino adulto. Registou-se uma morbidade e mortalidade médias de 13,75% e 4,22%, respectivamente. Os principais sinais clínicos foram emagrecimento progressivo, rigidez dos membros e claudicação. Foram realizadas necropsias, e os achados macroscópicos incluíram mineralização multifocal a coalescente de vasos sanguíneos, coração e pulmão. Microscopicamente, observou-se mineralização difusa grave, principalmente no endocárdio e nas camadas íntima e média da aorta. Observou-se mineralização intra-alveolar revestindo a parede dos septos alveolares, confirmando depósitos de sais de cálcio pela coloração de von Kossa. Os valores de cálcio e fósforo no sangue dos bovinos afetados permaneceram dentro dos intervalos de referência. O consumo de *S. glaucophyllum* foi confirmado em todos os surtos. Apesar de ser um problema endêmico na indústria de carne bovina na Argentina, não existem tratamentos ou estratégias de controle eficazes, portanto, mais estudos são necessários para prevenir a ocorrência de CE.

## Introduction

Enzootic calcinosis (EC) is a chronic toxic disease associated with the consumption of calcinogenic plants, affecting ruminants worldwide (Machado et al., 2020a). In South America EC is widely reported associated with the consumption of *Solanum glaucophyllum*, *Solanum stuckertii*, *Nierembergia veitchii* and *Nierembergia rivularis* (Riet-Correa et al., 2023). *S. glaucophyllum*, known as “duraznillo blanco”, is an endemic plant present in low-flooded areas, and the main cause of EC in Argentina, Brazil and Uruguay (García et al., 2017; Machado et al., 2020a; Riet-Correa et al., 2023). Particularly in Argentina, EC by *S. glaucophyllum* is among the most frequently diagnosed toxicities in cattle, producing severe economic losses in beef rearing systems (García et al., 2017). EC is characterized by hypercalcemia, hyperphosphatemia, hypoparathyroidism, hypercalcitoninism, soft tissue calcification, osteonecrosis and osteopetrosis (Machado et al., 2020a). Calcitriol (1,25(OH)_2_D_3_), an analogue of vitamin D_3_, is the main toxic principle of *S. glaucophyllum*, causing increased absorption of calcium (Ca) and phosphorus (P), leading mainly to systemic soft tissue mineralization (Jäpelt & Jakobsen, 2013). EC primarily occurs in livestock under extensive grazing systems due to the involuntary consumption of calcinogenic plants (Gimeno, 2000). However, EC outbreaks can also affect fattening animals when they consume hay heavily contaminated with *S. glaucophyllum* (Micheloud et al., 2012; Ovelar et al., 2024).

This retrospective study analyzes 23 outbreaks of EC in beef cattle caused by *S. glaucophyllum* consumption in central Argentina. It provides a detailed examination of epidemiological data, clinical presentations, and pathological findings, emphasizing common disease characteristics as well as rarer observations that warrant consideration.

## Case description

This retrospective study includes 23 outbreaks of EC caused by *S. glaucophyllum* in cattle registered from 1990 to 2024 by the Specialized Veterinary Diagnostic Service (SVDS) of INTA Balcarce. Diagnosis criteria was based on anamnesis, clinical signs, compatible gross and/or microscopic lesions, and evidence of consumption of *S. glaucophyllum*. Epidemiologic data were collected, including geographic location, animal’s age, production system, morbidity and mortality rates. In addition, clinical signs were recorded in all 23 outbreaks. A total of 30 necropsies were performed, though only in 13 tissue samples were fixed in 10% buffered formalin for 48 h and paraffin-embedded for histopathological analysis, including brain, cerebellum, spinal cord, heart, lung, liver, kidney, spleen, lymph nodes, brain, adrenal gland, skeletal muscle, pre-stomachs, abomasum, small and large intestine. Four micrometer sections were prepared routinely and stained with H&E. Selected lung, heart, and aorta sections were von Kossa stained for identification of Ca deposits.

Serum samples were collected from 54 affected animals of 10 outbreaks, to determine Ca (Cseh & Crenovich, 1996) and P levels (Cseh et al., 1994) by atomic absorption spectroscopy (AAS, Perkin Elmer AAnalyst 700, CT, USA). In all cases, the paddocks where the animals were grazing were inspected for identification of S. glaucophyllum. Also, the identification of other potential calcinogenic toxic plants from south America (*S. stuckertii, S. torvum, Stenotaphrum secundatum, Cestrum diurnum, Nierembergia riograndensis* and *N. rivularis*) were evaluated.

**Figure 1:**
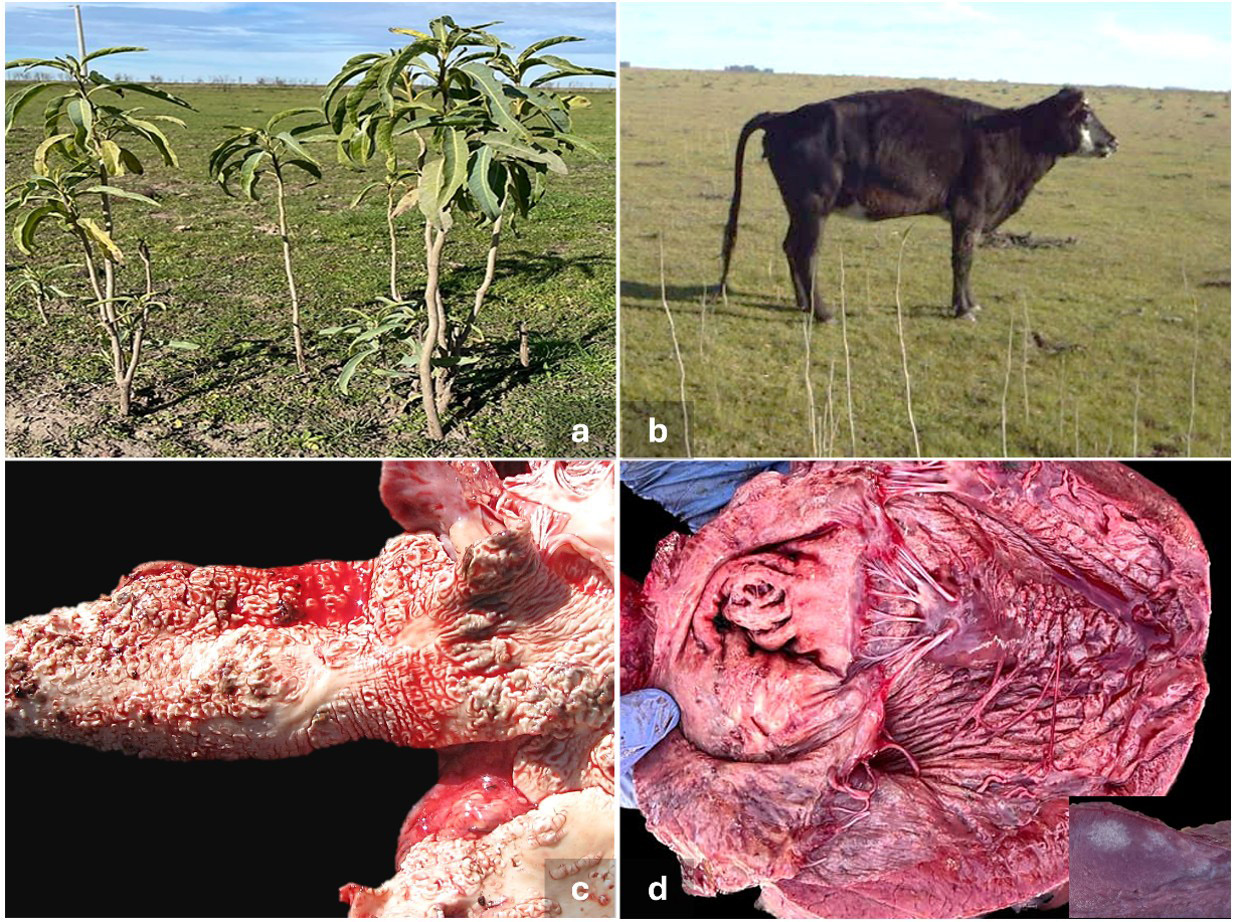
Epidemiological, clinical and macroscopic findings in an outbreak of enzootic calcinosis in cattle. **a)** *Solanum glaucophyllum* plants found in the pasture where the animals were grazing (outbreak #22). **b)** Heifer with progressive emaciation and notable kyphosis (outbreak #16). **c)** Severe diffuse mineralization of the aorta (outbreak #22). **d)** Rough, raised, whitish, protruding plaques, measuring from 0.1 to 6 cm long and 0.1 to 4 cm wide, due to mineral deposits in endocardium and heart valves (outbreak #14) Inset: multiple myocardial white delimited area in right ventricle papillary muscle.

Clinical EC outbreaks occurred throughout the whole year; however, a marked seasonality with 56.52% (13/23) outbreaks was registered between February and May (mid-summer to mid-autumn) (Table 1). All 23 EC outbreaks occurred in beef cattle (Table 1), affecting mainly breeding systems (78.2%; 18/23), 5 outbreaks in pasture fattening cattle (17.39%; 4/23) and one outbreak in *feedlot* cattle (4.34%; 1/23). Affected cattle were grazing mainly on native grasslands (21/23) and forage crops when the clinical signs were registered. The feedlot outbreak was previously reported by Micheloud et al. (2012) and affected steers that were consuming fescue hay contaminated with abundant amounts of leaves and whole plants of *S. glaucophyllum*. Adult cattle (greater than 2 years-old), mainly multiparous cows (11/23) were the category more frequently affected, followed by bulls (4/23). Young cattle (younger than 2 years-old) including heifers (5/23), steers (2/23) and calves (3/23; up to 9 months-old) were also affected (Table 1). In all outbreaks, *S. glaucophyllum* was identified as part of the grazing diet (**Figure 1A**), while in the animals confined in feedlots, the presence of *S. glaucophyllum* was observed in the fescue hay. In addition, other potential calcinogenic toxic plants from south America were not found. The herds affected in the outbreaks have an average of 221 animals (40 to 650), with a mean morbidity of 13.75% ± 9.33% (1.46% to 32.37%) and a mean mortality of 4.22% ± 3.53% (1.25% to 10.68%).

**Table 1:** Epidemiological, clinical and pathological data of 28 outbreaks of bovine enzootic calcinosis in central Argentina.

**Outbreak**	**Department**	**Month-Year**	**Categories affected**	**Feeding source**	**Production system**	**Clinicals signs**	**Gross findings**
1	Mar Chiquita	June-1993	Bull	Native grasslands	Breeding	NR	Calcification in large vessels, heart valves and lungs. Ascites
2	Mar Chiquita	May-1994	Cows/Heifers	Native grasslands and pastures	Breeding	progressive emaciation enophthalmia, diarrhea, osteophagy	Calcification in large vessels, lungs and tendons
3	General Madariaga	February-2000	Cows	Pastures	Breeding	progressive emaciation, stiff gait , xiphosis	Calcification in large vessels, lungs and tendons
4	General Madariaga	August-2000	Bulls	Pastures	Breeding	progressive emaciation and diarrhea	Calcification in large vessels, endocardium and lungs, ascites
5	Magdalena	April-2000	Calves	Pastures	Wintering	progressive emaciation, difficulty getting up , stiff gait	Calcification in large vessels and endocardium
6	Mar Chiquita	April-2004	Cows	Pastures	Breeding	NR	Calcification in large vessels and heart valves
7	Roque Perez	February-2006	Heifers	Native grasslands	Breeding	progressive emaciation, difficulty getting up , stiff gait	Calcification in large vessels and heart valves
8	Mar Chiquita	May-2006	Cows/Heifers	Native grasslands	Breeding	progressive emaciation, stiff gait	Calcification in large vessels, heart valves and lungs
9	Maipú	September-2006	Cows	Native grasslands	Breeding	difficulty getting up	Calcification in large vessels, heart valves and lungs
10	Maipú	February-2007	Cows	Native grasslands and pastures	Breeding	progressive emaciation, difficulty getting up , stiff gait	Calcification in large vessels
11	General Alvarado	April-2007	Heifers	Native grasslands	Breeding	progressive emaciation, stiff gait , xiphosis	Calcification in large vessels
12	Las Flores	February-2008	Heifers	Native grasslands	Breeding	progressive emaciation, stiff gait	Calcification in large vessels, heart valves and lungs
13	General Madariaga	April-2010	Steers	Native grasslands and pastures	Wintering	progressive emaciation, stiff gait , enophthalmia, osteophagy	Calcification in large vessels, heart valves and lungs
14	Saladillo	July-2010	Steers	Fescue hay and a starter ration	Feedlot	progressive emaciation, osteophagy	Calcification in large vessels, endocardium, heart valves and lungs. Whitish dots in renal cortex. Ascites
15	Mar Chiquita	May-2011	Cows	Pasture and ryegrass	Breeding	progressive emaciation, difficulty getting up , stiff gait	Calcification in large vessels, and heart valves
16	Saladillo	June-2011	Cows	Native grasslands	Breeding	progressive emaciation, difficulty getting up , stiff gait	Calcification in large vessels
17	Dolores	November-2011	Cows/Bulls	Pasture	Breeding	progressive emaciation, diarrhea	Calcification in large vessels, heart valves and lungs
18	Dolores	May-2013	Cows	Native grasslands	Breeding	progressive emaciation, xiphosis	Calcification in large vessels and endocardium
19	General Lavalle	July-2015	Calves	Native grassland, pasture and balanced feed	Wintering	difficulty getting up	Calcification in large vessels, endocardium and heart valves
20	Ayacucho	August-2016	Cows	Native grasslands	Breeding	difficulty getting up , sudden death	Calcification in large vessels. Whitish dots in renal cortex. Ascites.
21	Maipú	October-2019	Bulls	Native grasslands	Breeding	progressive emaciation	Calcification in large vessels and lungs. Ascites and hydrothorax
22	Coronel Suarez	June-2023	Calves	Native grasslands	Wintering	progressive emaciation, stiff gait , diarrhea, dyspnea	Calcification in large vessels, endocardium, heart valves, lungs, abomasum, rumen, meninges. Whitish dots in renal cortex
23	Tapalqué	April-2024	Cows	Native grasslands	Breeding	progressive emaciation, stiff gait , diarrhea	Calcification in large vessels, endocardium, heart valves, lungs and tendons

NR: no registered

Clinical signs were recorded in all 23 outbreaks, mainly characterized by progressive emaciation, in 100% of the outbreaks (23/23) (**Figure 1B**). In addition, stiff gait (50%) or difficulty getting up (46,15%) were frequently recorded. Other less frequent signs were kyphosis (4/23) (**Figure 1B**), diarrhea (6/23), dehydration (2/23), osteophagy (3/23), and dyspnea (1/23). In one outbreak cows were found dead without apparent previous signs.

Grossly in all 30 cases, systemic mineralization of the large vessels was observed, characterized by loss of elasticity and multiple to coalescing, elevated, white, rough plaques (**Figure 1C**). The carotid artery and aorta were the most severely affected. The heart was affected in 14 cases, with mineralization in valves (12/14) and/or endocardium characterized by multifocal to diffuse plaques (7/14) (**Figure 1D**). The atrioventricular and semilunar valves had focalized and well-defined mineralization characterized by the presence of hard and rough plaques. Rarely, mineralized foci were observed throughout the myocardium, with tendency to be more evident in papillary muscles (**Figure 1D**). Pulmonary calcification affecting the diaphragmatic lobes was observed in 8 cases, with the presence of multiple formations with a rough surface, measuring 1.5 to 2 cm in diameter, covering up to 50% of the affected lobe. Multifocal white mineralization streaks were observed throughout the renal cortex in three autopsies. Tendons were visibly affected in three cases, while only in one case transmural calcification in ruminal and abomasal mucosa and meninges was observed. Other gross findings were abundant ascites (5/21) and hydrothorax (1/21).

**Figure 2:**
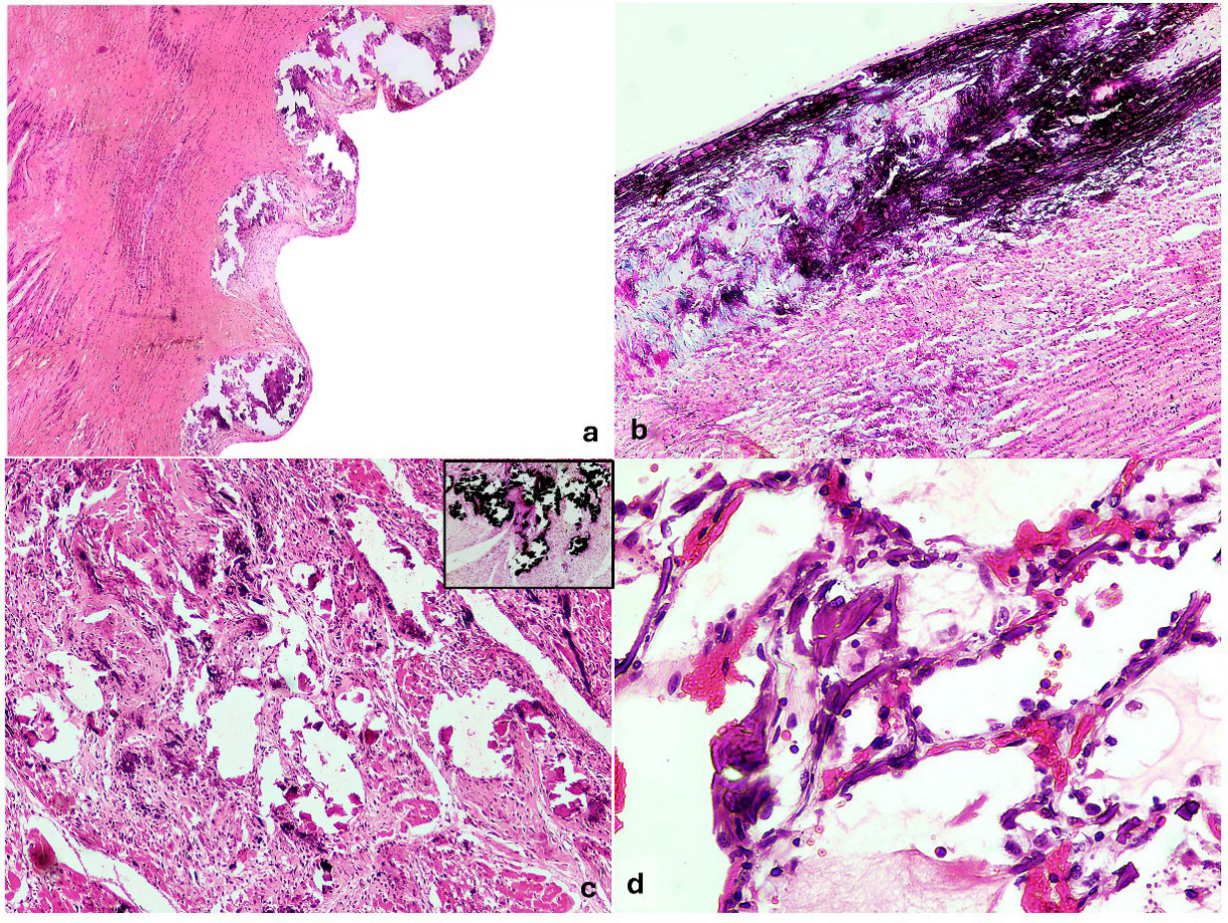
Microscopic findings in outbreaks of enzootic calcinosis in cattle. **a)** Aorta: severe multifocal-to-coalescent mineralization of the intima and media layers, resulted in protruding plaques towards the lumen. H&E, 40×. **b)** Aorta: severe diffuse mineralization of the intima and media layers. H&E, 200×. **c)** Heart: multiple areas in the myocardium (papillary muscle), extensive with a tendency to coalesce, of mineralization of myofibrils and replacement by severe fibrous tissue. H&E, 200×. Inset: abundant mineral deposits confirmed as calcium by Von Kossa stains, 200×. **d)** Lung: intra-alveolar mineralization lining the wall of the alveoli and alveolar septa with multifocal alveolar hemorrhage and edema. H&E, 400×.

Histological analysis was performed in 13 cases. Microscopically, severe diffuse mineralization of the intima and media layers (13/13) in the aorta was observed in all cases, while muscular arteries like carotid had nonmineralized hyperplasia of the intima layer and tunica media mineralization (8/13), including osteochondroid metaplasia (**Figure 2A**, B). Mineralization was characterized by irregular basophilic granular deposits. The multifocal-to-coalescent mineralization resulted in protruding plaques towards the lumen **(Figure 2A)**. In the heart, diffuse endocardial-subendocardial (3/13) and pericardial (1/13) mineralization and of adjacent vessels were observed (2/13), with mineralization of medium-caliber vessels in their media and intimal layers. Rarely (2/13), severe mineralization in the connective tissue of tendinous cords was observed. Additionally, multiple-to-coalescent areas of mineralization were present in the myocardium (papillary muscle), replacing cardiac myofibrils and were intermingled with fibrous tissue (**Figure 2C**), with myosatellite cells and angiogenesis were observed in one case. In the lung, intra-alveolar mineralization lining the wall of the alveoli and throughout alveolar septa most associated to capillary and small blood vessels walls (10/13) (**Figure 2D**) was present. This finding was rarely accompanied by multifocal alveolar hemorrhage (2/13), edema (2/13) and emphysema (3/13). In addition, segmental to complete mineralization of bronchial (2/13) and tracheal (1/13) cartilages were observed. Mineralization in other tissues including the renal arteries (intima and media layers) (1/13), multifocally the tubular epithelium, of the renal cortex (2/13) and medulla (4/13), the diaphragm serosa (1/13), spleen capsule (2/13), and the middle and intima layers of medium-caliber vessels of the spleen was present. The abomasum presented extensive mineralization in blood vessels (1/13) and muscular layer (2/13), with necrosis and ulceration of the mucosa (2/13). In one case, in rumen and reticulum, muscular arteries mineralization with severe necrosis of the serous and muscular layers, and necrotizing vasculitis was observed. In one case, the blood vessels of the meningeal membranes were mineralized. In lung, heart, and aorta sections, mineral deposits were confirmed as calcium deposits by von Kossa stains (**Figure 2C**).

From the 54 serum samples collected including affected animals of 10 outbreaks, mean Ca levels were within normal values (10.20 ± 1.19 mg/dl; range 4.97 to 13.7 mg/dl; reference value 8 to 12 mg/dl), and P serum levels (6.64 ± 1.96 mg/dl; range 2.7 to 12.89 mg/dl; reference value 3.5 to 7.5 mg/dl) were slightly to moderately elevated in 20 of the 54 samples (37.03%) (Kaneko et al., 2008).

## Discussion

This study describes the clinical and pathological findings in 23 outbreaks of EC due to *S. glaucophyllum* consumption in cattle in Buenos Aires province, Argentina. However, these outbreaks correspond to consultations by private veterinarians to the SVDS, being a much important problem, because the diagnosis is usually made in the field by practitioners or farmers and most cases are not reported to diagnostic laboratories. These EC outbreaks mainly affected beef cattle grazing in extensive production systems (García et al., 2017; Machado et al., 2020a). Though outbreaks were distributed throughout the year given its chronic characteristic, a seasonal pattern was observed from mid-summer to mid-autumn, possibly since the consumption of this plant mostly occurs in dry seasons or with little forage availability during winter and/or summer, leading the animals to consume on low grounds, being exposed to the plant or when leaves fall and mix with forage (Furlan et al., 2012; Gimeno, 2000). Multiparous cows were the most affected category, since they constitute the predominant population in the problem areas (Gimeno, 2000) and given by the prolonged stay in the farms, increasing exposition and consumption throughout their productive life could lead to the occurrence of clinical EC. In one case, poisoning occurred due to the consumption of contaminated hay, highlighting the toxicity of *S. glaucophyllum* for long periods after drying and with a subacute onset (Micheloud et al., 2012; Machado et al., 2020a; Ovelar et al., 2024). In most cases, progressive emaciation, difficulty getting up and stiff gait were observed (Gimeno, 2000; Machado et al., 2020a). EC is characterized by a chronic course with very noticeable nutritional and postural changes, being secondary to systemic mineralization (Machado et al., 2020a). In this sense, animals tend to remain recumbent and have a difficult time standing up and death can occur from extreme malnutrition and cachexia if animals are not removed from grazing paddocks with *S. glaucophyllum* (Furlan et al., 2012). These clinical signs only represent the last stage of the disease, having suffered the stages of metabolic alterations and anatomopathological lesions (Gimeno, 2000). The consumption of *S. glaucophyllum* produces an increase of intestinal absorption of Ca and P, and further blood increase within a few hours or up to 24 h after plant consumption (Majak, 2001; Machado et al., 2020a). In our study, the mean Ca and P levels were within the reference values, most probably because *S. glaucophyllum* consumption ceased days to weeks before the final clinical assessment. However, 37% of animals exhibited hyperphosphatemia, which may be linked to EC presentation. Another possible explanation is the naturally higher phosphorus levels in growing calves, as 12 of the 20 animals with hyperphosphatemia were calves. Additionally, the variance in phosphorus or calcium serum levels, is associated with transient changes of both minerals, rarely resulting in high levels during natural EC outbreaks (Machado et al., 2020a; Schild et al., 2021; Brown et al., 2024). Based on this, the diagnostic utility of serological values ​​of Ca and P in cases of natural poisoning by *S. glaucophyllum* seems very limited (Gimeno, 2000).

In all the outbreaks analyzed, pathological lesions produced by mineralization in soft tissues are similar to those reported in the literature (Furlan et al., 2012; Micheloud et al., 2012; Gimeno, 2000; Machado et al., 2020a). In our experience, mineralizations is a common incidental findings mainly in the aorta of many necropsied animals that have consumed *S. glaucophyllum*, probably affecting subclinical productive and reproductive parameters, though not severe enough to cause clinical signs and/or death. For the latter, widespread soft tissue mineralization is important. Aortic mineralization was a consistent finding in this study, along with medium- and large-caliber blood vessels throughout body, similar to previous reports in EC cases (Machado et al., 2020a; Schild et al., 2021). Less commonly, mineralization of small-caliber vessels in various organs has also been documented (Machado et al., 2020a), as observed in the spleen, kidney, pre-stomachs, lungs and heart. Additionally, calcification can extend beyond the arteries, affecting alveolar septa, renal tubules and cardiomyocytes (Barros & Rissi 2024). A direct action of the toxic principle of *S. glaucophyllum* on vascular smooth muscle cells was proposed to produce cellular dedifferentiation, bone and cartilage metaplasia and systemic mineralization of soft tissues (Machado et al., 2020a). Beyond this, decreases in the amount of collagen and elastic fibers present in the aorta were also observed in relation to the continuous consumption of *S. glaucophyllum* (Portiansky et al., 2002). All these changes may have an adverse influence on the cardiovascular function of the animals, resulting in hemodynamic changes like pulmonary edema and hydrothorax, and sudden death, commonly observed in sheep during management practices (Machado et al., 2020b). Additionally, it was shown that the extract of plants produces adverse effects on chondrocyte cultures from the epiphysis of the long bones of newborn rats (Bertassoli et al., 2023). Stiff gait was frequently observed in our study, a change that could be related to degenerative damage at the osteoarticular level.

It is recommended to avoid consumption of *S. glaucophyllum* based on rational grazing management and increase care in the preparation of silage or hay (Gimeno, 2000; Machado et al., 2020a). In addition, affected animals should not be exposed to stressful handling because they may develop cardiovascular failure (Machado et al., 2020a).

## Conclusions

Despite being a widely known problem, *S. glaucophyllum*-EC is still an endemic problem in the livestock industry in Argentina, generating great economic losses. For this reason, more studies are needed to increase in the understanding of the pathophysiological mechanisms to treat and/or prevent the adverse effects of EC. Also, further studies mainly focusing to subclinical and/or reproductive losses are needed, since the clinical observations of this study only represents the tip of the iceberg of the impact of EC to the economy of this sector.
